# Genetic landscape of T- and NK-cell post-transplant lymphoproliferative disorders

**DOI:** 10.18632/oncotarget.9400

**Published:** 2016-05-27

**Authors:** Elizabeth Margolskee, Vaidehi Jobanputra, Preti Jain, Jinli Chen, Karthik Ganapathi, Odelia Nahum, Brynn Levy, Julie Morscio, Vundavalli Murty, Thomas Tousseyn, Bachir Alobeid, Mahesh Mansukhani, Govind Bhagat

**Affiliations:** ^1^ Department of Pathology and Cell Biology, Columbia University Medical Center, New York, NY, USA; ^2^ Department of Pathology, Translational Cell and Tissue Research Laboratory, UZ Leuven/KU Leuven, Leuven, Belgium

**Keywords:** post-transplant lymphoproliferative disorders, immunodeficiency, genetic, genomic, T-cell lymphoma

## Abstract

Post-transplant lymphoproliferative disorders of T- or NK-cell origin (T/NK-PTLD) are rare entities and their genetic basis is unclear. We performed targeted sequencing of 465 cancer-related genes and high-resolution copy number analysis in 17 T-PTLD and 2 NK-PTLD cases. Overall, 377 variants were detected, with an average of 20 variants per case. Mutations of epigenetic modifier genes (*TET2, KMT2C, KMT2D, DNMT3A, ARID1B, ARID2, KDM6B*, *n*=11). and inactivation of *TP53* by mutation and/or deletion(*n*=6) were the most frequent alterations, seen across disease subtypes, followed by mutations of JAK*/*STAT pathway genes (*n*=5). Novel variants, including mutations in *TBX3* (*n*=3), *MED12* (*n*=3) and *MTOR* (*n*=1), were observed as well. High-level microsatellite instability was seen in 1 of 14 (7%) cases, which had a heterozygous PMS2 mutation. Complex copy number changes were detected in 8 of 16 (50%) cases and disease subtype-specific aberrations were also identified. In contrast to B-cell PTLDs, the molecular and genomic alterations observed in T/NK-PTLD appear similar to those reported for peripheral T-cell lymphomas occurring in immunocompetent hosts, which may suggest common genetic mechanisms of lymphoma development.

## INTRODUCTION

Post-transplant lymphoproliferative disorders (PTLD) encompass morphologically heterogeneous entities with variable clinical behavior. [1] PTLDs of B-lineage predominate, while T- and NK-cell PTLDs (T/NK-PTLD) are rare, representing 2-15% of all cases. [2] T/NK-PTLDs usually present late after transplantation, are associated with a poor prognosis, and, in contrast to B-cell PTLDs, only a minority are EBV-related (up to 37%). [2, 3]

The pathogenesis of T/NK-PTLDs and the underlying molecular alterations are presently unknown and their relationship to peripheral T-cell lymphomas (PTCL) arising in immunocompetent hosts remains unclear. Hence, in order to gain insights into the genetic bases of these uncommon PTLDs, we performed targeted next generation sequencing of cancer-associated genes and genome-wide DNA profiling to assess copy number alterations in a relatively large series of well characterized T/NK-PTLDs. We observed frequent mutations of epigenetic modifiers, members of the JAK/STAT signaling cascade, and TP53, and recurrent genomic alterations, similar to those described in PTCL arising in immunocompetent individuals.

## RESULTS

### Morphology and immunophenotype

**Figure 1:**
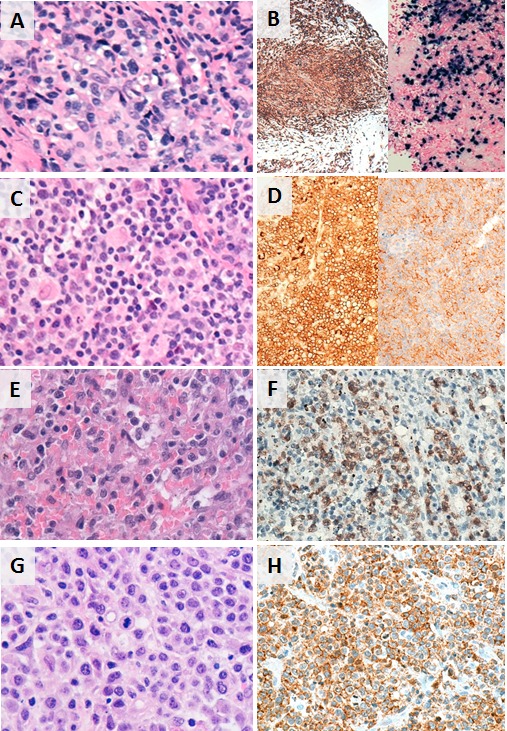
Histopathologic and phenotypic features of selected subtypes of T/NK-PTLD **A.** and **B.** EBV+ PTCL in the breast; CD8+ and EBER+. **C.** and **D.** PTCL, NOS presenting as a retroperitoneal mass; CD4+ and PD1+. **E.** and **F.** Hepatosplenic T-cell lymphoma in the spleen; TCR-gamma+. **G.** and **H.** PTCL, NOS in subcarinal lymph node; CD8+.

All T- and NK-cell lymphomas were classified based on morphologic and immunophenotypic features according to the 2008 WHO guidelines. [4] Immunophenotypic analysis was based on a combination of flow cytometry and immunohistochemistry. Peripheral T-cell lymphoma, not otherwise specified (PTCL, NOS) was the most common subtype (*n* = 6, 32%), followed by hepatosplenic T-cell lymphoma (HSTCL; *n* = 3), primary cutaneous anaplastic large cell lymphoma (c-ALCL; *n* = 3), angioimmunoblastic T-cell lymphoma (AITL; *n* = 2), extranodal NK/T-cell lymphoma, nasal type (ENKTCL; *n* = 2), EBV-positive peripheral T-cell lymphoma (EBV+ PTCL; *n* = 1), lymphomatoid papulosis (LyP; *n* = 1) and adult T-cell leukemia/lymphoma (ATLL; *n* = 1) (Table 1, representative cases are illustrated in Figure 1).

**Table 1:** Clinical, morphological and immunohistochemical data

Case	Age/Sex	Underlying dx	Graft	IS therapy	Time to PTLD (y)	Diagnosis	Location	CD2	CD3	CD5	CD7	CD4	CD8	CD56	TIA	CD30	EBER	Ki-67	Other	B-catenin	MLH1	MSH2	PMS2	MSH6	Gata3	Lineage	MSI status	TCR	Treatment	Status	Survival (days)
1	75F	Viral CM	Heart	AZA+CS+P	15.01	PTCL, NOS	BM	+	-	+	-	-	+	-	+	ND	-	30%		ND	ND	ND	ND	ND	ND	ND	MSS	Clonal	VMP	Dead	66
2	29M	Viral CM	Heart	AZA+CS+P	12.62	PTCL, NOS	Subcutaneous tissue, face	+	+	+	-	+	-	-	ND	-	-	ND	PD1-	-	+	+	+	-	-	ND	MSI-H	Clonal	CHOP/RT, gemcitabine	Dead	197
3	57M	Hypersensitivity pneumonitis	Left lung	CNI+MMF+P	0.47	PTCL, NOS	Subcarinal LN	+	+	+/−	−	−	+	−	+	−	−	80%	CD10-, CD15-, PD1-, CD25-, MUM1-	−	+	+	+	+	−	αβ	MSS	Clonal	EPOCH	Dead	98
4	75M	Dilated CM	Heart	CNI+MMF+P	2.00	PTCL, NOS	Retroperitoneum	+	+	+/−	+/−	+	−	−	−	−	−	40%	ALK-, BCL2-, MUM1-, PD1-	ND	ND	ND	ND	ND	ND	ND	ND	Clonal	Cytoxan/Etoposide	Dead	29
5	41F	Post-partum CM	Heart	CNI+P+MMF	7.01	PTCL, NOS	Retroperitoneum	+	+	+	−	+	−	−	−	−	−	ND	CD10-, BCL2+, PD1+, MUM1-	−	+	+	+	+	−	αβ	MSS	PC	EPOCH, GEMOX, Pralatrexate, Radiation and Auto-SCT	Alive	976
6	32F	CHF	Heart	CS	14.01	EBV+ PTCL	Breasts, bilateral	+	+	+	−	+/−	+	−	+	+	+	70%	CD10-, ALK-, MUM1-	ND	ND	ND	ND	ND	ND	ND	MSS	Clonal	EPOCH, Radiation and gemcitabine	Alive	1405
7	32M	N/A	Liver	CNI+AZA+CS	5.83	HSTCL	Spleen, liver, BM	+	+	−	+	−	+	+	+	−	−	ND		−	+	+	+	+		γδ	MSS	Clonal	VDMCT	Dead	120
8	34M	Dilated CM	Heart	AZA+CS+P	9.73	HSTCL	Spleen	ND	+	−	−	−	+	ND	+	−	−	ND		−	+	+	−	+	−	γδ	MSS	Clonal	CHOP, ESHAP, MNM	Dead	273
9	35M	GN, NOS	Kidney	AZA+CS+P	10.01	HSTCL	Spleen, liver	+	+	−	ND	−	−	+	+	ND	ND	ND	PD1-	−	−	+	+	+	70%	γδ	MSS	Clonal	CHOP	Dead	70
10	57F	DM	Kidney	n/a	24.43	AITL	Retroperitoneum	+	+	+	−	+	−	ND	ND	−	−	50%	BCL6+, CD10+, PD1+	−	+	+	+	+	30%	αβ	MSS	PC	RCHOP/Gemcitabine/Pralatrexate	Dead	321
11	44M	N/A	Lung	CNI+CS	4.08	AITL	LN, lung	+	+	+	+	+	−	−	ND	−	-*	ND	BCL6+, PD1+	ND	+	+	+	+	ND	ND	MSS	Clonal	Rituximab	Dead	730
12	83M	N/A	Kidney	CNI+MMF+CS	6.92	Primary cutaneous ALCL, CD30+	Skin, pleura	+	−	−	−	+	−	ND	−	+	−	ND	ALK-	−	+	+	+	+	ND	ND	MSS	ND	CHOP	Dead	120
13	47M	Nephrogenic systemic fibrosis	Kidney	CNI+MMF	4.73	Primary cutaneous ALCL, CD30+	Skin, back	+	−	−	−	+	−	ND	−	+	−	80%	ALK-, BCL2+	−	+	+	+	+	80%	αβ	MSS	Clonal	CHOP/RT	Dead	3839
14	71M	N/A	Heart	CNI+AZA+CS	18.00	Primary cutaneous ALCL, CD30+	Skin	+	−	−	−	+	−	ND	−	+	−	ND	ALK-	ND	ND	ND	ND	ND	ND	ND	ND	Clonal	CHOP	Dead	210
15	20F	Drug induced CM	Heart	AZA+CS+P	5.49	ENKTCL	Right breast	+	+	−	+	−	−	+	+	−	+	70%	CD25+	−	+	+	+	+	−	--	MSS	PC	N/A	Dead	18
16	71F	Hypersensitivity pneumonitis	Lung	CNI+AZA+P	7.40	ENKTCL	Nasal vestibule, lung	+	+	+	+/−	−	+	−	+	−	+	90%		ND	ND	ND	ND	ND	ND	αβ	ND	Clonal	None	Dead	61
17	43F	FSGS	Kidney	CNI+AZA+P	6.98	ATLL	Retroperitoneum	+	−	−	−	+	−	−	ND	+	−	ND	ALK-, FoxP3-, CD25+, MUM1+	ND	ND	ND	ND	ND	ND	ND	ND	Clonal	EPOCH X4	Alive	650
18	83M	APCKD	Kidney	CNI+MMF	3.23	Lymphomatoid papulosis	Skin, left forearm	+	−	−	−	−	−	−	ND	+	−	ND	CD15+, ALK-, MUM1+	ND	ND	ND	ND	ND	ND	ND	ND	PC	Reduced IS	Alive	2741
19	70M	N/A	Kidney	CNI+AZA+CS	1.92	PTCL, NOS	Pleura, pericardium, LN	+	+	−	−	+	−	−	−	−	−	ND	ALK-	ND	ND	ND	ND	ND	ND	ND	MSS	Clonal	None	Dead	60

Immunosuppression; MSI: Microsatellite instability; TCR: T-cell receptor clonality; CM: Cardiomyopathy; CHD: Congestive heart failure; GN: Glomerulonephritis; DM: Diabetes Mellitus; FSGS: Focal segmental glomerulosclerosis; APCKD: Autosomal dominant polycystic kidney disease; LN: Lymph node; BM: Bone marrow; AZA: Azathioprine; CS: Corticosteroids; P: Prednisone; CNI: Calcineurin inhibitor; MMF: Mycophenolate mofetil; RT: Radiotherapy; N/A: Not available; ND: Not done; PC: Polyclonal;

* EBER+ B-cells were present

PTCL, NOS (*n* = 6) comprised CD4+ and CD8+ cases with downregulation or loss of CD7 seen in all. One CD8+ T-cell lymphoma expressed CD30 and showed evidence of EBV infection by *in situ* hybridization for EBER. The three cases of HSTCL were derived from TCR-gamma-delta T-cells. They expressed TIA-1 but not the cytotoxic granule constituents (granzyme-B, perforin) and lacked CD5 expression. The AITL cases were CD4+ and displayed expanded and disrupted follicular dendritic cell meshworks. One case had a concomitant EBV+ clonal B-cell proliferation. The c-ALCL cases showed diffuse dermal infiltrates of large CD30+ T-cells, many exhibiting prominent nucleoli, and lacked ALK expression. Both ENKTCL were EBV+. One was of cytotoxic T-cell origin involving the breast and the other of NK-cell origin involving the nasal vestibule and lung; both displayed cellular atypia, had high proliferation indices and showed angiodestruction and extensive necrosis. The ATLL presented as lymphomatous disease and the neoplastic cells expressed CD4 and CD25.

### Clinical characteristics

The detailed clinical features of the 19 T/NK-PTLD are described in Table 1. The median age was 47 (range: 20-83) and 12/19 (63%) patients were male. The most commonly transplanted organ was heart (*n* = 8), followed by kidney (*n* = 7), lung (*n* = 3) and liver (*n* = 1). Immunosuppressive therapy comprised calcineurin inhibitors (*n* = 12), corticosteroids (*n* = 11), azathioprine (*n* = 10), and mycophenolate mofitil (*n* = 6); no association between the type of therapy and organ allograft was observed. The median interval between organ transplantation and diagnosis was 7 years (range: 0.5 - 18 y). Extranodal disease was seen in 15/19 (79%) cases and 3 (16%) cases were EBV+. The types of therapies for PTLD and response to treatment varied. The patient with lymphomatoid papulosis (case 18) was treated with reduction in immunosuppression alone, which led to disease regression. Four patients received a combination of chemotherapy and radiation and 12 received chemotherapy alone. Two patients were referred for palliative care. Clinical remission was achieved in 1 patient with c-ALCL (case 13) who presented with localized disease, after treatment with chemotherapy and radiation; he died of disease unrelated causes 12 years post diagnosis. One patient with EBV+ PTCL (case 6) is in remission 4 years after diagnosis having received chemotherapy with etoposide, prednisolone, vincristine, cyclophosphamide, and doxorubicin (EPOCH) and one individual with PTCL, NOS (case 5) achieved remission after chemotherapy, radiation, and allogeneic stem cell transplantation. The patient diagnosed with ATLL, based on positive serology for HTLV-1, was treated with EPOCH and is alive 2 years after diagnosis. Despite aggressive treatment, however, the median overall survival was 6.6 months (range: 18 days to 12 years).

### Genome-wide copy number alterations

Copy number alterations were observed in 12/16 cases (75%; complex = 8, simple = 4). Loss of heterozygosity was noted in 7/16 (44%) cases (Figure 3F; Table 2). No recurrent alterations were seen in 5 cases of PTCL, NOS. Two of three HSTCL showed 7q gain and 7p loss, indicative of isochromosome 7q. One of two ENKTCL showed loss of 6q. Copy number alterations in 1 of 2 cases of c-ALCL analyzed included 1p gain and losses at 13q and 16q. No copy number changes were detected in the ATLL and lymphomatoid papulosis.

**Table 2:** Genomic alterations in T/NK-PTLDs

Case	Subtype	Gain/Loss	Locus	Base Positions	Size (mb)	Total Genes	Genes identified in Cancer Gene Census
1	PTCL, NOS	Not analyzed					
2	PTCL, NOS	Loss	7p22.1p15.3	chr7:6,327,460-23,472,983	17.14Mb	85	1(ETV1)
3	PTCL, NOS	Gain	9p24.3p24.1	chr9:681,960-7,237,233	6.5mb	42	2 (JAK2, CD274)
		Gain	11q22.3q25	chr11:105,115,252-135,006,516	29.89	304	11 (ATM, DDX10, POU2AF1, SDHD, PAFAH1B2, PCSK7, MLL, DDX6, ARHGEF12, FLI1)
		Loss	6q11.1q27	chr6:62,173,195-170,837,508	108.66	561	10 (PRDM1, ROS1, GOPC, STL, MYB, TNFAIP3, ECT2L, EZR, FGFR1OP, MLLT4)
4	PTCL, NOS	Loss	20q13.13q13.2	Chr20:48,080,029-51,759119	3.42Mb	33	None
5	PTCL, NOS	Gain	4q28.1q34.1	chr4:125,081,373-175,395,062	50.31Mb	180	19(FBXW7)
		Gain	14q31.1q32.33	chr14:83,163,335-106,495,970	23.33Mb	294	8 (TRIP11, GOLGA5, DICER1, TCL6, TCL1A, BCL11B, AKT1, IGH)
		LOH	10q23.32q25.1	chr10:92,990,108-106,337,119	13.34	196	3 (TLX1, NFKB2, SUFU)
		Loss	17p13.3p11.2	chr17:400,959-21,516,719	21.11Mb	419	6 (YWHAE, USP6, TP53, PER1, GAS7, MAP2k4)
6	EBV+ PTCL	Gain	2q14.2q37.3	chr2:120,196,122-243,199,373	123.00Mb	803	13(ERCC3, CHN1, HOXD13, HOXD11, NFE2L2, PMS1, SF3B1, CREB1, IDH1, ATIC, FEV, PAX3, ACSL3)
		Gain	3p26.1p14.3	chr3:7,153,652-58,391,565	51.23Mb	528	12(SRGAP3, FANCD2, VHL, PPARG, RAF1, XPC, MLH1, MYD88, CTNNB1, SETD2, BAP1, PBRM1)
		Loss	6q11.2q24.2	chr6:57,730,420-143,488,404	85.75Mb	370	7(PRDM1, ROS1, GOPC, STL, MYB, TNFAIP3, ECT2L)
		LOH	17q21.2q25.3	chr17:40,324,798-80,263,427	39.93Mb	678	14(BRCA1, ETV4, COL1A1, HLF, MSI2, CLTC, BRIP1, CD79B, DDX5, PRKAR1A, SRSF2, CANT1, ASPSC, STAT3)
		LOH	9q33.3q34.13	chr9:126,794,173-135,302,624	8.5	144	4 (SET, FNBP1, ABL1, NUP214)
		LOH	15q23q25.1	chr15:71,689,059-79,548,077	7.85	121	1 (PML)
		Loss	Xp22.33q28	chrX:177,942-155,219,364	155.04Mb	1285	CRLF2, P2RY8, ZRSR2, BCOR, KDM6A, SSX1, WAS, GATA1, TFE3, SSX2, KDM5C, MSN, MED12, NONO, ATRX, SEPT6, ELF4, GPC3, PHF6, MTCP1)
7	HSTCL	Gain	1q21.3 - q25.2	chr1:154,087,033-176,562,796	22.47576	369	7 (TPM3, MUC1, PRCC, NTRK1, SDHC, FCGR2B, PBX1)
		Loss	4p15.32 - p15.2	chr4:16,152,859-25,102,176	8.949318	29	None
		Loss	4q13.1 - q23	chr4:62,726,234-100,740,709	38.01448	230	1 (RAP1GDS1)
		Loss	7p22.2 - p11.1	chr7:3,896,634-61,064,518	57.16789	373	9 (PMS2, ETV1, HNRNPA2B1, HOXA9, HOXA11, HOXA13, JAZF1, IKZF1, EGFR)
		Gain	7q11.22 - q21.11	chr7:70,468,676-77,724,388	7.255713	103	2 (ELN, HIP1)
		LOH	7q31.1 - q36.3	chr7:110,440,775-159,118,443	48.7	401	7 (MET, SMO, CREB3L2, KIAA1549, BRAF, EZH2, MLL3)
		Gain	8q11.21 - q24.3	chr8:51,192,818-146,364,002	95.2	571	10 (TCEA1, PLAG1, CHCHD7, NCOA2, HEY1, COX6C, EXT1, MYC, NDRG1, RECQL4)
		LOH	11q13.3q25	chr11:70,834,389-135,006,516	64.2	559	15 (NUMA1, PICALM, MAML2, BIRC3, ATM, DDX10, POU2AF1, SDHD, PAFAH1B2, PCSK7, MLL, DDX6, CBL, ARHGEF12, FLI1)
		Gain	17q21.31-q25.3	chr17:41,516,675-80,366,217	16.8	634	12 (ETV4, COL1A1, HLF, MSI2, CLTC, BRIP1, CD79B, DDX5, PRKAR1A, SRSF2, CANT1, ASPS)
		Gain	22q12.3 - q13.33	chr22:32,252,434-51,304,566	19.1	302	4 (MYH9, PDGFB, MKL1, EP300)
		Homozygous loss	Yp11.31 - q11.23	chrY:2,660,163-28,799,935	26.1	189	None
8	HSTCL	Loss	7p22.3p11.2	chr7:41,421-55,272,715	55.23Mb	396	8(CARD11, PMS2, ETV1, HNRNPA2B1, HOXA9, HOXA11, HOXA13, JAZF1, IKZF1, EGFR)
		Gain	7q11.21q36.3	chr7:61,816,116-159,118,443	97.3	838	12(SBDS, ELN, HIP1, AKAP9, CDK6, MET, SMO, CREB3L2, KIAA1549, BRAF, EZH2, MLL3)
9	HSTCL	No changes identified.					
10	AITL	Loss	1p31.3 - p31.1	chr1:66,994,889-80,226,910	13.2	72	1 (FUBP1)
		Loss	1p13.3 - p11.2	chr1:109,527,108-121,350,934	11.0	151	5 (RBM15, TRIM33, NRAS, FAM46C, NOTCH2)
		Loss	2q11.1 - q14.1	chr2:95,439,005-116,163,121	20.7	210	2 (TTL, PAX8)
		Loss	2q14.1 - q23.1	chr2:117,810,213-149,194,919	31.4	138	1 (ERCC3)
		LOH	4p16.3 - q35.2	chr4:71,566-190,915,650	190.8	1034	13 (FGFR3, WHSC1, SLC34A2, PHOX2B, FIP1L1, PDGFRA, CHIC2, KIT, KDR, RAP1GDS1, TET2, IL2, FBXW7)
		Gain	5q31.1 - q31.2	chr5:130,788,642-138,657,896	7.9	102	None
		Loss	5q31.2 - q32	chr5:138,689,223-149,136,632	10.4	165	None
		Loss	5q33.3 - q34	chr5:157,096,900-160,315,446	3.2	25	1 (EBF1)
		Gain	5q35.1 - q35.2	chr5:170,275,961-172,901,400	2.6	24	3 (RANBP17, TLX3, NPM1)
		Gain	6p25.3 - p25.1	chr6:204,909-5,172,009	5.0	48	1 (IRF4)
		LOH	6p25.3 - p21.1	chr6:5211336-45699405	40.5	628	10 (DEK, HIST1H4I, TRIM27, POU5F1, DAXX, HMGA1, FANCE, PIM1, TFEB, CCND3)
		Gain	7p22.3 - p15.3	chr7:41,421-22,798,650	22.8	159	3 (CARD11, PMS2, ETV1)
		Gain	7q21.11 - q31.31	chr7:80,054,945-119,098,598	39.0	317	3 (AKAP9, CDK6, MET)
		Gain	7q36.2 - q36.3	chr7:154,693,849-157,479,833	2.8	26	None
		Loss	8p23.3 - p23.1	chr8:172,417-7,004,147	6.8	47	None
		Gain	8q22.2 - q24.11	chr8:99,374,877-117,703,509	18.3	66	1 (COX6C)
		Gain	9q21.11 - q34.3	chr9:71,043,202-141118911	70.1	730	15 (GNAQ, SYK, OMD, FANCC, XPA, NR4A3, TAL2, SET, FNBP1, ABL1, NUP214, TSC1, RALGDS, BRD3, NOTCH1)
		Gain	11q13.4 - q25	chr11:71,703,483-134,938,847	63.2	543	15 (NUMA1, PICALM, MAML2, BIRC3, ATM, DDX10, POU2AF1, SDHD, PAFAH1B2, PCSK7, MLL, DDX6, CBL, ARHGEF12, FLI1)
		LOH	16p13.3 - p13.2	chr16:83,887-8,065,382	8.0	235	2 (TSC2, CREBBP)
		LOH	17p13.3 - p11.2	chr17:400,959-16,277,578	15.9	319	6 (YWHAE, USP6, TP53, PER1, GAS7, MAP2K4)
		Loss	17q24.3 - q25.3	chr17:67,876,646-76,799,898	8.6	145	1 (SRSF2)
		Loss	17q25.3	chr17:79,372,489-80,263,427	0.9	44	1 (ASPSCR1)
		Gain	22q12.1 - q12.3	chr22:27,148,121-35,540,423	8.4	100	4 (MN1, CHEK2, EWSR1, NF2)
		Gain	Xp22.33	chrX:177,942-2,000,942	1.8	15	2 (CRLF2, P2RY8)
		Loss	Xp22.2	chrX:10,983,386-12,957,293	2.0	9	None
		LOH	Xp22.33 - p11.1	chrX:177,942-155,219,364	155.0	1286	21 (CRLF2, P2RY8, ZRSR2, BCOR, KDM6A, SSX1, SSX4, WAS, GATA1, TFE3, SSX2, KDM5C, MSN, MED12, NONO, ATRX, SEPT6, ELF4, GPC3, PHF6, MTCP1)
		Loss	Xp21.3 - p11.3	chrX:28,468,750-43,844,600	15.4	57	1 (BCOR)
		Gain	Xp11.3 - p11.22	chrX:43,851,171-53,446,146	9.6	221	8 (KDM6A, SSX1, SSX4, WAS, GATA1, TFE3, SSX2, KDM5C)
		Loss	Xq21.31 - q22.3	chrX:90,213,826-105,763,052	15.5	90	None
		Loss	Xq23 - q25	chrX:112,406,196-121,321,850	8.9	162	1 (SEPT6)
		Loss	Xq27.3 - q28	chrX:143,811,457-155,219,364	11.4	209	1 (MTCP1)
11	AITL	No changes identified.					
12	c-ALCL	Gain	6q21-q24.1	chr6:109,262,907-142,278,857	33.0	30	2 (ROS1, GOPC)
		LOH	17p13.3-12	chr17:1-15,645,289	15.6	315	6 (YWHAE, USP6, TP53, PER1, GAS7, MAP2K)
13	c-ALCL	Gain	1p36.22p36.21	chr1:9,744,244-13,187,252	3.44Mb	72	None
		Gain	10p15.3p12.2	chr10:126,070-23,745,727	23.61Mb	160	2(GATA3, MLLT10)
		Loss	13q13.1q34	chr13:32,540,047-115,103,150	82.56Mb	438	5(BRCA2, LHFP, LCP1, RB1, ERCC5)
		Loss	16q12.1q23.2	chr16:49,691,925-80,146,038	30.45Mb	303	6 (CYLD, HERPUD1, CDH11, CBFB, CDH1, MAF)
		Loss	16q23.3q24.1	chr16:82,431,694-86,293,870	3.86Mb	41	None
14	c-ALCL	Not analyzed					
15	ENKTCL	Gain	2q24.3q32.3	chr2:164,197,701-195,534,597	31.33 Mb	183	5 (CHN1, HOXD13, HOXD11, NFE2l2, PMS1)
		LOH	2q33.1-q37.3	chr2:198,858,917-243,052,331	44.19Mb	401	6 (CREB1, IDH1, ATIC, FEV, PAX3, ACSL3)
		Loss	3p13p12.3	chr3:69,852,242-79,214,617	9.36	29	2(MITF, FOXP1)
		Loss	4q32.3q35.2	chr4:166,281,785-190,915,650	24.6	98	None
		Gain	5q21.3q35.3	chr5:105,294,321-180,698,312	75.4	62	9 (APC, PDGFRB, CD74, ITK, EBF1, RANBP17, TLX3, NPM1, NSD1)
		Gain	6p25.3p24.3	chr6:204,909-9,047,746	8.84Mb	67	1 (IRF4)
		Loss	6q16.3q23.3	chr6:101,280,508-138,764,089	37.43	204	None
		Gain	9q13q34.12	chr9:68,170,421-133,962,930	65.79	559	10 (GNAQ, SYK, OMD, FANCC, XPA, NR4A3, TAL2, SET, FNBP1, ABL1)
		Loss	12q24.33	132,663,422-133,818,115	1.15kb	23	None
		Loss	15q24.1q26.2	chr15:73,313,691-97,999,066	24.68Mb	281	5(PML, NTRK3, IDH2, CRTC3, BLM)
		Loss	17p13.3p11.2	chr17:400,959-20,301,297	19.90Mb	403	7 (YWHAE, USP6, TP53, PER1, GAS7, MAP2K4, STAT3)
		Loss	17q24.1q24.2	chr17:62,680,553-64,867,456	2.1Mb	179	None
		Loss	17q11.2q25.3	Chr17:25,899,925-79,934,193	54.09Mb	1034	21 (NF1, SUZ12, TAF15, MLLT6, LASP1, CDK12, ERBB2, RARA, BRCA1, ETV4, COL1A1, HLF, MSI2, CLTC, BRIP1, CD79B, DDX5, PRKAR1A, SRSF2, CANT1, ASPSCR1)
		Loss	18p11.32q23	Chr18:128,557-77,991,543	77.86Mb	382	4 (ZNF521, SS18, MALT1, BCL2)
		Loss	19p13.3	chr19:247,232-2,303,276	2.0MB	92	3(FSTL3, STK11, TCF3)
		Gain	Xq27.3q28	chrX:146,155,369-155,219,364	9.06Mb	195	1(MTCP1)
16	ENKTCL	Not analyzed					
17	ATLL	No changes identified.					
18	LYP	No changes identified.					
19	PTCL, NOS	Gain	1q21.1 - q32.1	chr1:144,940,761-206,669,136	61.7	859	15 (PDE4DIP, BCL9, ARNT, TPM3, MUC1, PRCC, NTRK1, SDHC, FCGR2B, PBX1, ABL2, TPR, MDM4, ELK4, SLC45A3)
		LOH	3p26.3 - p21.1	chr3:63,411-52,826,707	52.8	510	12 (SRGAP3, FANCD2, VHL, PPARG, RAF1, XPC, MLH1, MYD88, CTNNB1, SETD2, BAP1, PBRM1)
		Loss	7p22.3 - p11.2	chr7:1-57,754,465	58.0	422	10 (CARD11, PMS2, ETV1, HNRNPA2B1, HOXA9, HOXA11, HOXA13, JAZF1, IKZF1, EGFR)
		Gain	7q11.21-q34	chr7:62827731-142378708	79.6	658	10 (SBDS, ELN, HIP1, AKAP9, CDK6, MET, SMO, CREB3L2, KIAA1549, BRAF)
		Gain	8p23.3q24.3	chr8:172,417-146,292,734	146.1	966	15 (WHSC1L1, FGFR1, HOOK3, TCEA1, PLAG1, CHCHD7, PCM1, WRN, NCOA2, HEY1, COX6C, EXT1, MYC, NDRG1, RECQL4)

LOH: Loss of heterozygosity

### Targeted next generation sequencing

**Figure 2:**
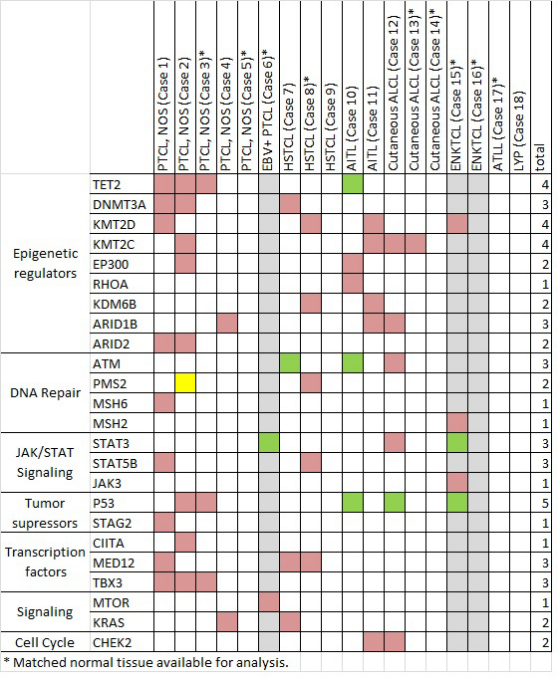
Selected mutations identified by next-generation sequencing in 18 cases of T/NK-PTLD Total number of subjects with mutations is listed at right. A green box indicates mutation co-occurring with genomic loss or LOH, yellow box indicates MSI-H status as a consequence of mutation and grey columns indicate 3 EBV+ PTLD.

Somatic mutations were detected in 17/18 cases (94%) using targeted sequencing of 465 cancer associated genes. Overall, 377 variants were identified: 286 missense (76%), 31 indels (8%), 16 nonsense (4%), and 44 intronic (12%) (Suppl. Table 1), with an average of 594.8-fold coverage and > 10X coverage for 99% of the coding regions (Suppl. Table 2). The pathogenic variants, focusing on genes recurrently targeted or implicated in hematologic malignancies, are listed in Figure 2.

Heterozygous mutations of epigenetic modifier genes were the most frequently observed alterations (11/18, 61%). *TET2* mutations were seen in four cases; missense (*n* = 2, including R1359G), frameshift (*n* = 1), and nonsense accompanied by LOH of 4q24 (*n* = 1, R544X). Mutations in histone and DNA methyltransferases (*KMT2D*, *KMT2C, DNMT3A*) and the histone demethylase (*KDM6B*) occurred in 7/18 (39%) cases. Missense and frameshift mutations in chromatin remodeling complex genes (*ARID1B*, *ARID2*) were seen in 5 (28%) cases.

**Figure 3:**
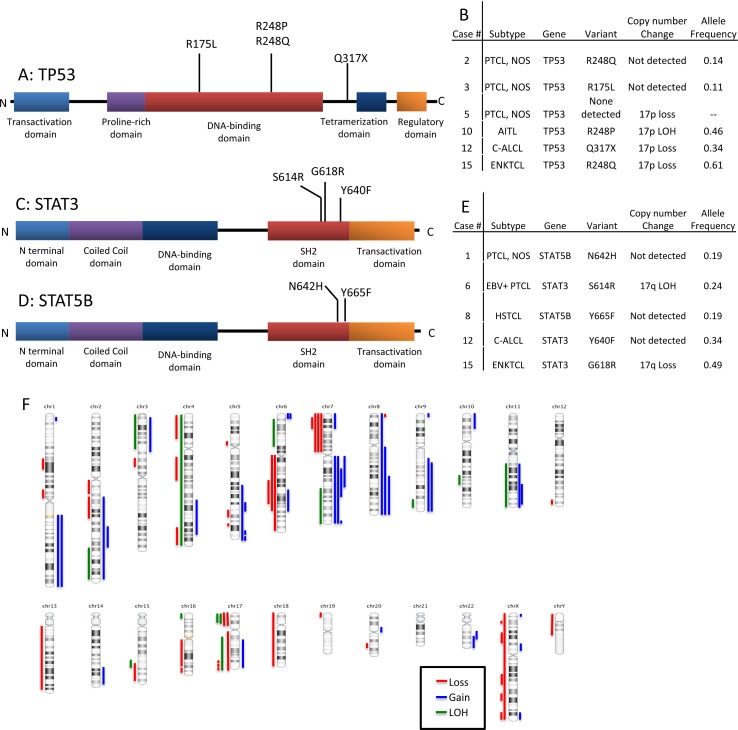
Schematic representation of the types and locations of *TP53, STAT3* and *STAT5b* mutations **A.** *TP53* mutations. **B.** Table showing T/NK-PTLD subtypes with *TP53* mutation and 17p copy number changes. **C.** *STAT3* mutations in the SH2 domain. **D.** *STAT5b* mutations in the SH2 domain. **E.** Table showing T/NK-PTLD subtypes with *STAT3* or *5b* mutations, and 17q copy number changes. **F.** Ideogram of copy number changes detected in 16 cases of T/NK-PTLD. Gains are indicated in blue, losses in red and LOH in green.

Disruption of *TP53* was identified in 6 of 18 (33%) cases (Figure 3A, 3B). Biallelic inactivation was noted in 3 cases (co-occurrence of mutation with 17p LOH) and monoallelic missense mutations in two cases. All missense mutations (*n* = 4) are classified as non-functional or partially functional in the IARC *TP53* database (Version R17). [5] Deletion of 17p encompassing the *TP53* locus alone was seen in one case.

Alterations of JAK/STAT pathway genes were seen in 5 of 18 cases (28%). *STAT3* and *STAT5B* mutations were mutually exclusive (Figure 3C-3E). STAT5B mutations were present in one case each of HSTCL and PTCL, NOS. *STAT3* mutations were present in one case each of c-ALCL, ENKTCL, and the EBV+ PTCL. LOH of the STAT3 locus on 17q was observed in the two EBV+ PTLDs with *STAT3* mutations (EBV+ PTCL and ENKTCL), suggesting biallelic gain of function. These two cases also had loss of 6q encompassing *FOXO3*, *PRDM1* and *PTPRK*. A *JAK3* variant (S568P) adjacent to a previously described hotspot was also present in the *STAT3*-mutated EBV+ PTCL. [6]

Subtype-specific genetic alterations were also identified. One of the two cases of angioimmunoblastic T-cell lymphoma demonstrated known pathogenic mutations in *RHOA* and *FYN,* proteins involved in T-cell motility and proliferation and TCR signaling. [7]

Importantly, mutations in genes not previously implicated in PTCL pathogenesis were also observed. Missense mutations in *MED12* were seen in 3 cases (1 PTCL, NOS and 2 HSTCL), including two novel and potentially damaging variants (R516H and R816Q) and one exon 2 mutation (L36P). Alterations of *TBX3* were observed in 3 of 5 PTCL, NOS. A missense mutation in *MTOR* (W1456L) involving the FAT domain was observed in the EBV+ PTCL. A three base pair insertion mutation was observed in *FBXW7* in one PTCL, NOS, which was confirmed by Sanger sequencing (Suppl. Figure 1).

### Microsatellite instability

Variants in mismatch repair (MMR) genes were detected in 4 of 18 PTLD (cases 1, 2, 8, and 15, Figure 2). However only one (6%) PTLD (case 2) with a heterozygous *PMS2* mutation and PMS2 loss by immunohistochemistry showed evidence of high level microsatellite instability (MSI-H); alterations at 5/5 mononucleotide repeats accounting for 7% (1/14) of all cases analyzed with a panel of microsatellite markers. The remaining 3 cases showed normal immunohistochemical expression of the MMR gene products.

## DISCUSSION

Our series of T/NK-PTLD comprised a spectrum of PTCL subtypes that have been reported to occur post organ transplantation and are recognized as monomorphic PTLDs in the 2008 WHO classification [3, 4]. Epidemiologic data drawn from national registries have shown a higher frequency of PTCL, NOS, ALCL, HSTCL, and ENKTCL in transplant recipients. [8] Data regarding other PTCL subtypes in the setting of immunosuppression (e.g. AITL, ATLL) are limited. [9, 10] Cases of ATLL, however, have been reported in recipients of solid organ allografts in multiple series. [11, 12] In fact, immunosuppression has been proposed as a risk factor for neoplastic transformation in HTLV-1 carriers post organ transplantation. [13] Our observations support the broad definition of T/NK-PTLD in the current WHO classification and suggest inclusion of more detailed phenotypic information in PTLD databases, which will allow precise classification of PTCL and help determine the role of immune deregulation in the pathogenesis of different disease subtypes.

The clinicopathological features of T/NK-PTLD are distinct from B-cell PTLDs. In contrast to B-cell PTLDs, which are EBV-driven in 55-65% of cases, only 3 of 19 (16%) T/NK-PTLD in our series showed evidence of EBV infection. [14] Consistent with prior reports, disease presentation was late after transplant, with a median of 7 years between onset of immunosuppression and the development of lymphoma. The time of occurrence of B-cell PTLDs has an apparent bimodal distribution with the largest peak occurring in the first year after transplantation and a smaller peak observed five years after transplantation. [8] While a subset of B-cell PTLDs respond to reduction in immunosuppression, the majority of the T/NK-cell PTLDs do not, and they are associated with poor outcomes despite aggressive treatment. [15] Disease biology, however, appears to mirror the subtype specific differences observed in T-cell lymphomas of immunocompetent individuals. Lymphomatoid papulosis followed a fairly indolent course with survival over 10 years, while ENKTCL and HSTCL were rapidly fatal.

The genomic alterations detected in our cohort were similar to those described in PTCLs occurring in immunocompetent hosts. [16, 17] Changes consistent with isochromosome 7q, a characteristic feature of HSTCL, were identified in 2 cases of HSTCL. Loss of 13q, encompassing *ING1* and 16q, encompassing the *CTCF* locus, seen in one c-ALCL, have been reported in non-transplant associated c-ALCL. [18] One of two ENKTCL and the EBV+ PTCL showed loss of 6q. This recurrent abnormality, which is observed in 29-52% of ENKTCL arising in immunocompetent individuals, encompasses loci of the *FOXO3* and *PRDM1* genes, which have been shown to function as tumor suppressors in ENKTCL and subtypes of PTCL. [16, 19, 20] In contrast to EBV+ B-PTLD, however, we observed a higher frequency of complex copy number changes in EBV+ T-PTLD. [21]

Mutations of epigenetic modifier genes were the most frequent alterations (61%) in T/NK-PTLD and were most commonly observed in cases of PTCL, NOS and AITL. A similar mutational spectrum is frequently observed in PTCL occurring in immunocompetent individuals. [7, 22, 23] Loss of function mutations in *KMT2D*/*MLL2*, a lysine specific histone methyltransferase targeting H3K4 residues that is considered to function as a tumor suppressor, have been reported in PTCL arising in immunocompetent individuals and were seen in a variety of T/NK-PTLD. [24] Missense mutations in *TET2*, a methylcytosine dioxygenase that plays an important role in active DNA demethylation, are frequent in both PTCL, NOS and AITL (38% and 33-76%, respectively). [23, 25, 26] Loss of function mutations in *DNMT3A*, a DNA methyltransferase that is required for genome-wide de novo methylation, are frequent in a variety of malignancies and have been reported in up to 38% of PTCL, NOS and 33%-37% AITL. [23, 26, 27] In a murine model, the combination of *TET2* loss and *DNMT3A* mutation appeared to cooperate in the development of T-cell lymphomas exhibiting features of AITL. [28] Similar to observations in PTCL, NOS and AITL occurring in immunocompetent individuals, concomitant *TET2* and *DNMT3A* mutations were noted in two cases of PTCL, NOS in our series. [29] Mutations in chromatin modifier genes, including *ARID1B*, and *ARID2*, were also common, a finding in line with prior studies of PTCL occurring in immunocompetent hosts. [24]

The TP53 pathway is frequently dysregulated in a variety of neoplasms, most commonly by *TP53* mutation or loss, and these lesions are associated with aggressive disease. [30] *TP53* abnormalities are known recurrent changes in a variety of PTCL, albeit at a low frequency (8-9%) and their prognostic significance is unclear in these neoplasms. [31–33] The higher frequency of *TP53* aberrations in our series (33%) could reflect the small sample size and future larger studies are warranted to determine if the frequency of this genetic lesion is higher in PTCL arising in states of immune dysregulation.

Alterations of the JAK/STAT pathway are frequent in diverse subtypes of PTCL of immunocompetent hosts, including ALCL, HSTCL, T-PLL, ENKTCL, and also in LGL. [6, 34–37] Genetic aberrations activating the JAK-STAT pathway were identified in 28% of T/NK-PTLD, including PTCL, NOS, HSTCL, c-ALCL, and ENKTCL, and an EBV+ PTCL. As previously observed in PTCL, *STAT3* and *STAT5b* alterations were mutually exclusive in T/NK-cell PTLD as well. [38] We observed an association between EBV infection and STAT3 activation. *STAT3* mutations and LOH of the *STAT3* locus were seen in 2 of 3 EBV+ T/NK-PTLDs with concomitant loss of 6q encompassing *PTPRK.* Loss of PTPRK has been associated with constitutive STAT3 activation in ENKTCL. [39] Activation of STAT3 by EBV,** even in the absence of genetic lesions, has been shown to induce lymphoproliferative disorders of T- and NK-cells. [40] EBNA-2 is a coactivator of *STAT3* transcription and LMP1 is known to constitutively activate STAT3 in B-cells. [41, 42] Of interest, IFN-gamma induced by LMP1 has been shown to enhance STAT1 expression in EBV+ B-PTLD. [43] Whether analogous mechanisms are operational in EBV-associated T/NK-PTLD is not yet known. Nonetheless, our findings suggest that targeting the JAK-STAT pathway with currently available drugs or more selective agents might be of therapeutic benefit in a subset of T/NK-PTLD.

Mutations in genes not previously described in PTCL were also observed. Novel and potentially damaging variants in *MED12* were observed in two cases and a *MED12* exon 2 mutation (L36P), recently described in chronic lymphocytic leukemia, was seen in one case. [44] MED12 is part of the CDK8 subcomplex, which includes CDK8 kinase and cyclin C, of Mediator, a component of the preinitiation complex, with integral roles in transcriptional regulation. [45] The MED12 protein has been shown to regulate JAK/STAT signaling in mouse embryonic fibroblasts [46]. The role of MED12 in normal and neoplastic T-cells, however, is unclear. The functional consequence of mutations of the transcription factor T-box 3 (*TBX3*), a transcriptional repressor, important for myriad developmental processes, [47] noted in three cases, is also not known at present. A novel missense mutation in *MTOR* was detected in an EBV+ PTCL. Similar mutations, which have been shown to activate both mTORC1 and 2 signaling in other neoplasms, may contribute to T-cell growth, survival and proliferation, in part by metabolic reprogramming of lymphocytes. [48] Activation of the mTOR pathway has been reported in T-cell lymphomas, but the presence of mTOR mutations has not been systematically investigated in these malignancies. Of interest, rapamycin analogues, which disrupt the mTORC signaling cascade have shown promise in early phase clinical trials of PTCL patients. [49–51]

Azathioprine use has been associated with an increased risk of hematological malignancies in allograft recipients and individuals with autoimmune diseases. [52] Thiopurines have been implicated in MMR pathway defects possibly due to epigenetic silencing of the MMR enzymes. [53] Borie et al. reported microsatellite instability (MSI-H) in 9% of immunosuppression-related lymphomas, including 33% (6/18 cases) of PTCL post solid organ transplantation. [52] Only 1 of 10 patients in our series treated with an azathioprine-based immunosuppressive regimen developed a MSI-H tumor. Although the observed frequency of MSI-H was lower in our series (1/14 cases; 7%) than a prior study, this difference was not statisically significant (*p* = 0.1).

In summary, our study represents the first survey of the genetic landscape of T/NK-PTLDs and adds to the growing list of genes altered in T-cell neoplasms that affect a variety of T-cell functions, including activation, proliferation, epigenetic regulation and metabolism. The observed clinicopathologic features and genetic aberrations appear similar to those reported for PTCL subtypes occurring in “immunocompetent” individuals, suggesting shared pathogenetic mechanisms. Our findings raise the possibility of novel therapeutic options for these aggressive lymphomas that need to be explored further in pre-clinical studies.

## MATERIALS AND METHODS

### Case selection

The archives of the Departments of Pathology, Columbia University Medical Center, New York and University Hospitals of KU, Leuven were searched for cases of T/NK-PTLD. Nineteen cases were identified (Columbia University Medical Center, *n* = 14; University Hospitals of KU, Leuven, *n* = 5) that had adequate formalin-fixed, paraffin-embedded (FFPE) tissue for analysis (> 50% tumor, except for one case of lymphomatoid papulosis with a tumor burden of 20%). We defined T/NK-PTLD as T- or NK-cell lymphomas occurring in individuals after organ transplantation, in accordance with the WHO 2008 guidelines. Morphologic and phenotypic data were reviewed and the lymphomas were classified according to current WHO criteria. [1] Patient demographic and clinical data were collected from the electronic medical record. This study was approved by the Institutional Review Board of Columbia University and performed in accordance with the Declaration of Helsinki.

### Whole genome copy number and LOH analysis

Genome-wide copy number and LOH analysis was performed for 16 cases using the Affymetrix OncoScan FFPE assay (Affymetrix, CA, USA). Briefly, the Oncoscan assay has been optimized to work with DNA from FFPE tissue, utilizing molecular inversion probes to identify changes in copy number and loss of heterozygosity. [54] DNA extraction, sample preparation, hybridization, and scanning were performed according to the manufacturer's specifications. Analysis was performed using the Affymetrix Chromosome Analysis Suite 2.0 (ChAS) and Nexus Copy Number 7.5 software (Biodiscovery, Inc. CA, USA).

All copy number alterations and regions of LOH called by the software were verified manually to determine erroneous calls and identify clonal gains and losses undetected by the software(s). Analysis was restricted to gains and losses > 1 Mb in length. Genomic alterations are reported based on the NCBI build 37 (hg19) of the human genome and cancer-associated genes were curated from the Cancer Gene Census (COSMIC v61 Release;http://www.sanger.ac.uk/genetics/CGP/Census/).

### Next generation sequencing analysis

Targeted next generation sequencing of 18 tumors and 9 matched normal samples was performed using a panel comprising 465 cancer-associated genes (Suppl. Table 3). Fifty to 250 ng of DNA, extracted using the Qiamp mini kit or the Qiamp FFPE kit (Qiagen, Germantown, USA) was fragmented to a median of 150-200bp, by sonication. Following end-repair and 3′ adenylation of the fragments, and ligation of double-stranded sequencing and indexing adaptors to both ends, target capture and enrichment was performed with the Sure Select Hybrid Capture system (Agilent Technologies, Santa Clara, USA), using custom designed probes. Libraries were then quantified using qPCR, diluted to 2nM and pooled, prior to cluster generation and analysis on Illumina HiSeq2500, using Illumina TruSeq v3 chemistry (San Diego, USA) and 100bp paired-end reads (up to 9 indexed samples per run). Fastq files (more than 70% of reads with > Q30) were demultiplexed with CASAVA, and samples with at least 6Gb of sequencing data were used for mapping and variant calling using NextGene Software (Softgenetics, State College, USA). Reads were aligned to the hg19 (GRCh37) reference genome. At least 3 variant reads were required to call a variant. All calls with ambiguous alignments were excluded, including calls corresponding to regions with large indels. Candidate variants at very low allele frequency (< 10% of variant reads) were eliminated.

After annotation, the variants were cross referenced with those in the 1000 Genomes Project, OMIM, dbSNP, and the Exome Variant Server. Variants with an allele prevalence > 1% in the 1000 genomes project were excluded. Common variants present in our departmental database of variants identified in prior constitutional exome analysis, non-pathogenic variants reported in dbSNP, and low quality calls were filtered out. The remaining variants were submitted for manual curation and variant prioritization with visual review of alignments. Synonymous variants and intronic variants greater than 2 bp from the coding sequence were excluded. Variants were manually cross-referenced with the Catalog of Somatic Variants in Cancer (COSMIC) and those that were not known “hot spot” mutations or had not been previously reported as potential driver variants were analyzed by PROVEAN and SIFT algorithms. For cases with matched normal specimens, variants present in the normal samples were excluded.

### Assessment of microsatellite instability

Microsatellite instability (MSI) testing was performed in 14 cases using a fluorescent PCR-based assay (MSI Analysis system, Version 1.2, Promega, Madison, WI). Briefly, this test assessed 5 mononucleotide repeats (BAT25, BAT26, NR-21, NR-24, and MONO-27) and 2 pentanucleotide repeats (PentaC and PentaD) on genomic DNA and matched normal, where available. The fluorescently labeled PCR products were analyzed by capillary gel electrophoresis. MSI was determined if the tumor alleles showed a size difference ≥3 bp. Tumors with 2 or more microsatellite unstable markers were classified as MSI-H; the remainder were classified as microsatellite stable (MSS). Immunohistochemistry was performed for MLH1 (clone M1, predilute, Ventana), MSH6 (clone 44, predilute, Ventana), MSH2 (clone G219-1129, predilute, Cell Marque) and PMS2 (clone EPR3947, predilute, Cell Marque) using an autostainer (Benchmark Ultra^TM^ Ventana, Tuscon, AZ) after heat induced antigen retrieval (EDTA, pH 7.3). The ultraView^TM^ Universal DAB Detection kit (Ventana) was used for visualization. Absence of nuclear staining was evaluated in lesional tissue; adjacent stromal cells were used as an internal control.

Methods for DNA extraction, immunohistochemistry, flow cytometry, and Sanger sequencing are described in the supplemental methods.

## SUPPLEMENTARY MATERIALS FIGURES AND TABLES








