# Supplementary material for: Genetic landscape of T- and NK-cell post-transplant lymphoproliferative disorders
Source: Oncotarget. 2016 May 27;7(25):37636–48. doi: 10.18632/oncotarget.9400 (PMC5122338; doi:10.18632/oncotarget.9400)
Supplement: Supplementary file 1 [file oncotarget-07-37636-s001.pdf]

Genetic landscape of T- and NK-cell post-transplant lymphoproliferative disorders

Supplemental Methods

Morphological and Immunophenotypic Analysis

Immunohistochemical (IHC) stains were performed on formalin-fixed, paraffin-embedded (FFPE) tissue sections after moist heat-induced antigen retrieval, using an autostainer and commercially available primary antibodies, according to standard methods. *In situ* hybridization was performed for EBV-encoded small RNAs (EBER) according to the manufacturer's protocol.

Four-color flow cytometric analysis was performed on fresh tissue (FACScan; BD Bioscience, San Jose, CA) using a comprehensive panel of antibodies to evaluate cell lineage. Data were analyzed with Cell Quest software (BD bioscience).

DNA Extraction and Quantification

Ten sections, each 10 mm thick, were taken of the archived FFPE tissue blocks used for clinical diagnosis. When available, DNA was extracted from normal tissues from the same patient. There was no microdissection of the FFPE specimens. DNA was extracted and purified using the Qiagen kit following the manufacturer's recommended protocol. Extracted DNA was quantified using Quant-iT PicoGreen ds DNA HS reagent and Qubit fluorometer (Invitrogen, Carlsbad, California) following the manufacturer's protocol.

Sanger Sequencing

Recurrent mutations deemed to be pathogenic were confirmed by Sanger sequencing according to standard methods. Briefly, PCR products obtained with specific primers flanking the mutation of interest were treated with exonuclease and shrimp alkaline phosphatase to remove remaining primers and dNTPS, and used as substrates for cycle sequencing using the BidDye Terminator Version 3.1 chemistry (Thermo Fisher, Springfield Township, USA), analyzed on an ABI 3130XL capillary sequencer (ThermoFisher, USA), and evaluated using Mutation Surveyor (Softgenetics, State College, USA).

Supplemental results

We confirmed two variants (FBXW7, NM_001257069.1, c.45_46insCCT, p.T15_G16insP variant in case 5 (Supplemental Figure 1) and TBX3 NM_016569.3, c.1280G>A, R427Q in case 3) by conventional Sanger Sequencing assay in the two cases with available material for analysis.

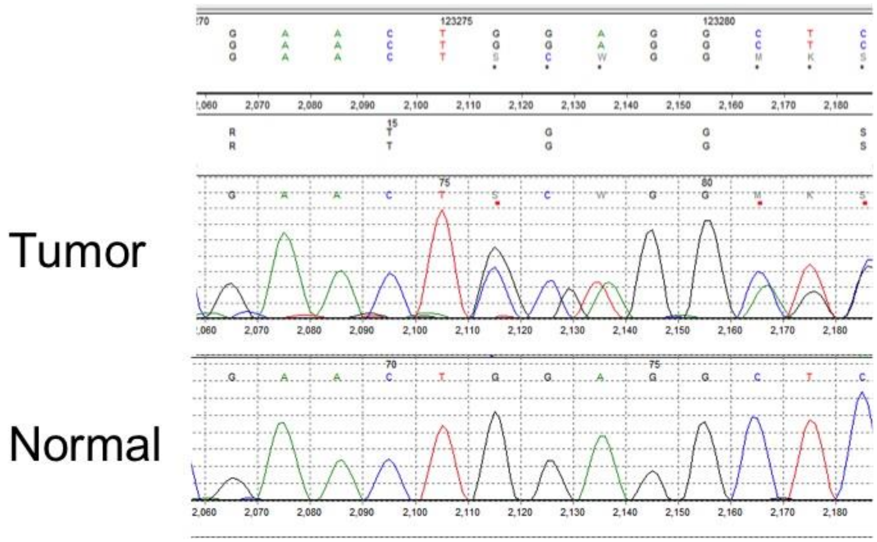

Supp. Figure 1: Sanger confirmation of FBXW7 variant (c.45_46insCCT; p.T15_G16insP) in case 5.
